# Variation of local zoological knowledge about Southern river otter and other semi-aquatic mammals in Nahuel Huapi National Park (Argentina)

**DOI:** 10.1186/s13002-023-00590-8

**Published:** 2023-05-09

**Authors:** Carla M. Pozzi, Ana H. Ladio

**Affiliations:** 1 Área Biología de la Conservación, Programa de Estudios Aplicados a la Conservación del Parque Nacional Nahuel Huapi (CENAC), Depto. de Conservación y Educación Ambiental – Parque Nacional Nahuel Huapi, San Carlos de Bariloche, Río Negro Argentina; 2 Instituto de Investigaciones en Biodiversidad y Medio Ambiente (INIBIOMA) CONICET, Universidad Nacional del Comahue, San Carlos de Bariloche, Argentina; 3 Grupo de Etnobiología, Universidad Nacional del Comahue, San Carlos de Bariloche, Río Negro Argentina

**Keywords:** Citizen science, Ethnozoology, Participatory workshops, Otter

## Abstract

**Background:**

The huillín *(Lontra provocax,* Thomas 1908) is an otter, endemic to southern Argentina and Chile. It is in danger of extinction. In the Nahuel Huapi National Park and surroundings is the only freshwater, known and stable population of huillín in Argentina. In this park, several urban and rural centers coexist with this species. The main objective of our work was to answer: How does the local zoological knowledge (LZK) vary about the huillín, particularly its identification and sighting, among people from different social groups, with different ages and gender, who live in the rural or urban environment and with different periods of permanence in the place?

**Methods:**

Ninety-six written interviews were conducted using visual stimuli to ensure that interviewees refer to the huillín. In addition, we also inquire about the LZK of other species with which it can be confused. Additional open interviews were conducted with participants who observed the huillín to determine the georeferencing of the reported sites and include them on a final map.

**Results:**

95% of people identified the huillín and this was confused with the coipo in 3% and with the american mink, in 5%. The results show that, in general, the LZK did not vary significantly with the sociocultural characteristics of the participants, showing a remarkable homogeneity. However, people in rural areas are more likely to observe the species than people in urban areas. Moreover, people between 20 and 40 years of age are more likely to observe the huillín. The LZK mapping has identified areas that are consistent with and/or adjacent to official source records. Other areas have also been identified that may provide new information.

**Conclusion:**

With this participatory work, we realize that the species is recognized by urban and rural inhabitants and very few confused it. The homogeneity in the LZK found constitutes a kick for the realization of other participatory studies that promote lines of research, action and management that improve the quality of the environments where the only freshwater, known and stable population of the huillín in Argentina lives.

**Supplementary information:**

The online version contains supplementary material available at (10.1186/s13002-023-00590-8).

## Background

The huillín *(Lontra provocax)* is an endangered otter endemic to southern Argentina and Chile [1, 2]. In Argentina, the species has a distribution restricted to two distant population centers: the upper basin of the Limay River (freshwater) and the marine coasts of the Fuegian Archipelago (Beagle Channel, Mitre Peninsula and Isla de Los Estados) [1]. The huillín is the “top predator” of the aquatic systems of this area. Animals are so called when they prey on others, but are not themselves preyed on by any other animals; they are at the top of the food pyramid, controlling the abundance and dynamics of the food chain [3, 4]. Otter species are considered indicators of the state of integrity of the ecosystem, being the first to disappear when the environment suffers alterations, due to their high location in the trophic chain [5]. For this reason, the huillín is considered an "umbrella species" since through its conservation the care of the entire aquatic ecosystem can be promoted [6].

According to various authors, the current distribution of the huillín is a consequence of the hunting pressure suffered by the species due to the fur industry [7–9]. It has been suggested that the current distribution of huillín in freshwater has been reduced by 80%. [10]. The only known stable freshwater population for Argentina is in Northern Patagonia, in and around Nahuel Huapi National Park (PNNH for its acronym in Spanish) [9–11]. For this reason, the huillín is the emblem of PNNH and has been declared a species of special value for this organization as well as for the municipality of San Carlos de Bariloche, one of the most important cities bordering the protected area.

San Carlos de Bariloche, Villa La Angostura and the smaller towns of Villa Traful are examples of urban centers that coexist directly or indirectly with the huillín. These cities and towns are characterized by being relatively young localities, with a history of no more than 120 years; their human population is heterogeneous in terms of origins and cultural identities, including both original Mapuche settlers whose communities pre-existed in the area as well as internal and external immigrants. However, so far there are no studies that account for people's Local Zoological Knowledge (LZK) about this emblematic otter species.

This situation of lack of information is paradoxical given the urgent need for conservation of the huillín because of its very low area of occupancy (348 km^2^ for Argentina) as well as its vulnerability due to fragmented populations and a continuous decrease in the quality of its habitat [1, 2] In this sense, conservation organizations warn of the need to include local communities and their knowledge in order to articulate joint strategies that generate empathy and care for the species [12–14].

Links with animals are learned by people through their direct experience with the environment and/or by different mechanisms of cultural transmission [15]. All these aspects are part of LZK, defined from ethnobiology as people's knowledge about animals as part of their cultural heritage, which varies according to the influence of different ecological and sociocultural factors [16]. The combination of a set of characters, mainly morphological (color-shape, that is, visual stimuli), sensory, ecological and utilitarian ones, among others, are those that allow the identification of animal species among people [17].

Numerous studies show that LZK varies according to age [18–21], gender [18, 20–23] and whether the socio-environmental context of individuals is rural or urban [18, 24, 25]. In addition, it has been found that it varies with the years of permanence in a given place, because people gain experience of contact with the animals that live there [21, 22].

Faunal species are often confused with each other because: (a) they have similar morphological or ethological features and/or (b) they are named in the same way in the same locality. Such is the case of solenodon (*Solenodon paradoxus,* Brandt 1833) and hutia (*Plagiodontia aedium,* Cuvier 1836), two species of micromammals with similar traits that can be confused with each other and with non-native small mammals (e.g., rats, mongooses, guinea pigs; [26, 27]. The study by [28] on the Caribbean island La Española shows that both species are named by the local communities with the same common name “jutia” generating misunderstandings in their recording.

In South America, there are two species of animals commonly known as otters: the lutrinos, which belong to the group of mustelids (Order Carnivora), and the coipo, which are rodents (O. Rodentia) [29]. In northern Patagonia, the huillín (belonging to the lutrino group) and the coipo inhabit the same areas and are confused with each other because they share the same name “nutria” [4, 30, 31]. In addition, another semi-aquatic mammal inhabits next to both species: the american mink (*Neovison vison,* Schreber 1777). This species is exotic and invasive in its Patagonian distribution. Originally from North America, Alaska, Canada and most of the United States, it was introduced in our country in 1935 to be bred to obtain its pelts for the fur industry [32]. Later, releases and escapes from its farms led to the establishment of wild populations both on the continent and in Tierra del Fuego [10]. The first animals, in wild state, were observed in the 1960s, and since then the species has been expanding [10]. The huillín can be confused because it has similar ethological and morphological traits to the american mink and the coipo.

In the last two decades, the participation of civil society in different aspects of scientific research has been encouraged in what is called citizen science. According to [33], this is a type of scientific production based on the conscious and voluntary participation of citizens that allows the maximization of objectives, activities and knowledge [34]. It represents a participatory research model that involves the public in scientific projects, usually in data collection and, in some cases, in the collective interpretation of results [35]. It is important to consider how the institutions involved as well as the unique characteristics of the natural resource or species being monitored influence the methods, results and appropriateness of public participation in citizen science [36]. One of the most interesting aspects of the purpose of this tool is its intention to mobilize knowledge between scientists and citizens in order to solve complex problems collectively [37].

In this sense, citizen science and ethnozoology would offer a space for appropriate integration to act on endangered species, as is the case of the huillín, as well as other otter species [38–40]. Therefore, the main objective of our work was to answer: How does LZK about the huillín, particularly its identification and sighting, vary among people of different social groups in PNNH, with different ages and gender, who live in rural or urban areas, and with different length of residence? Our predictions are that the highest proportion of people who recognize the huillín are natives of the area, older people, and people with a longer period of residence. Regarding gender, no predicted direction has been proposed.

In addition, given that the characters employed by people in the description of a species provide a store of information about the characters that cultures employ when they identify and/or classify it [17], we studied how the huillín is named in local taxonomies, and/or confused with the native coipo and the exotic american mink. Additionally, we analyzed whether the identification of the native coipo and the exotic american mink varies in the same way in relation to the variables mentioned above. Finally, with the information collected together with citizens, we made a collective map of huillín sighting sites in order to incorporate probable sighting sites and to discuss the scope of its conservation in PNNH and surrounding areas.

### Study area

**Fig. 1:**
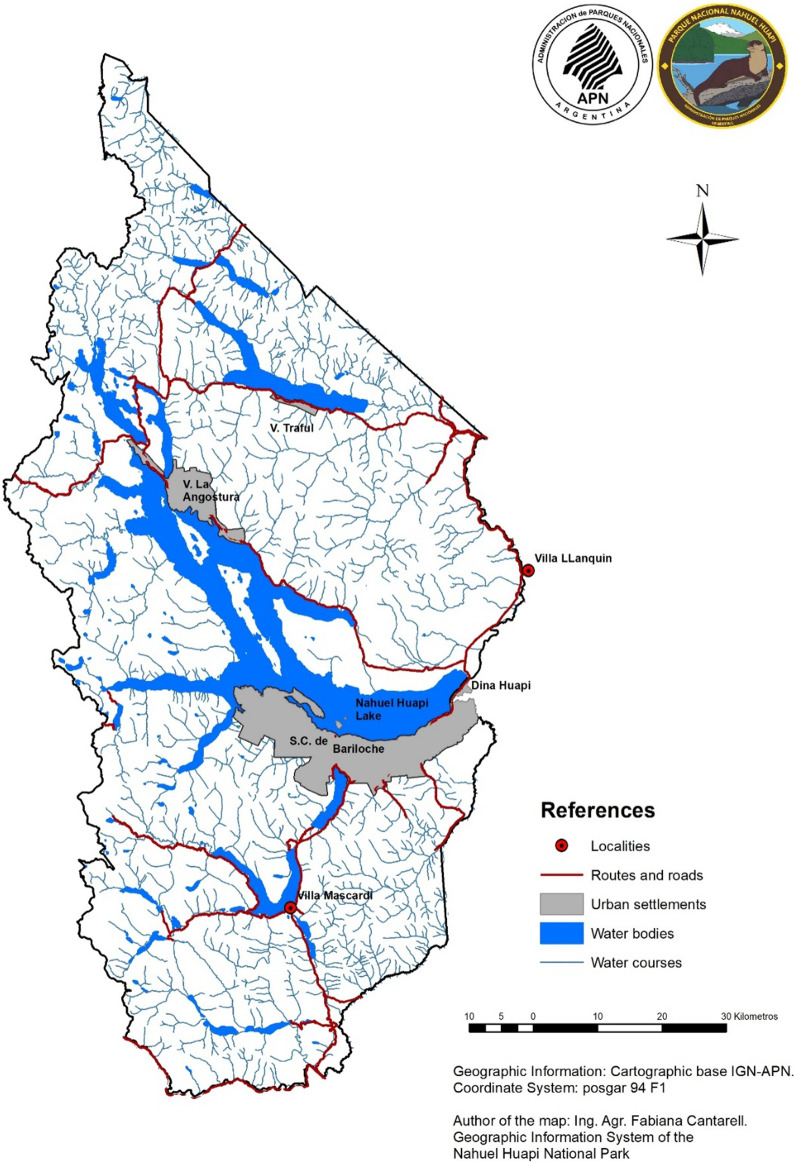
Map of Nahuel Huapi National Park and urban settlements bordering the protected area

PNNH has an area of approximately 717,261 ha. The altitudinal range of the area is 400–3480 m.a.s.l. The average annual temperature is 10º C, and the annual range of rainfall varies between 500 and 2000 mm. Rainfall is mainly concentrated during winter in the form of snow. Summers are hot and dry [41]. The protected area is bordered by important cities such as San Carlos de Bariloche (Rio Negro, 112.000 inhabitants), Villa La Angostura (Neuquén, 11.000 inhabitants) and Villa Traful (Neuquén, 417 inhabitants) [INDEC (Instituto Nacional De Estadística & Censos), 2010], as shown in Fig. 1. These urban centers are expanding and inducing population growth and the strengthening of the tourism profile of the region, mainly in the case of San Carlos de Bariloche and Villa La Angostura, although the rest of the populated areas are heading in the same direction. The largest city in terms of surface area and number of inhabitants is San Carlos de Bariloche (41°09′ S/71°18′ O), considered the fastest growing in the last decades in Argentina [42]. In general terms, its urbanization process has had a disorderly and poorly planned character, which currently generates numerous environmental problems, some of them difficult to solve. Examples of this lack of foresight are the occupation of flood-prone areas (flood plains of rivers, streams and mallines) and the poor choice of sites for waste disposal, with the consequent contamination of water and soil [43]. The accelerated real estate pressure and its consequent threat to forests and watersheds is one of the most critical conservation problems faced by the huillín in this city [44].

The region is multicultural, consisting of members of the Mapuche people as well as Chilean citizens, European immigrants and Argentinian citizens. Since the colonization of the Argentine State (late nineteenth century) in this region of pre-existence of indigenous people, the area experienced a significant increase in population due to immigration and a remarkable process of urbanization [45].

## Materials and methods

### Organization of workshops

This research was based on two steps. First, between 2015 and 2018, six participatory workshops were held (Additional file 1: Table Sa) with people linked in various ways to PNNH. These workshops were part of the updating tasks on wildlife issues that the institution carries out with different sectors of society and the agency. The workshops were used as an opportunity to consult about people's LZK, specifically about the semi-aquatic mammals that inhabit Northern Patagonia and, in particular, about the huillín. They were attended by PNNH tour guides, park rangers from the protected area as well as from neighboring provincial areas and neighbors from San Carlos de Bariloche, and were announced through the media and invitations to neighborhood councils or development associations, so participation was open and free. The participants who finally attended were people concerned and interested in the care of the coasts, flora and fauna of semi-aquatic environments who lived and/or worked near and/or in the distribution area of the huillín. Prior informed consent was requested from all participants in accordance with the Ethnobiological Code of Ethics (ISE 2006) [46]. Before the interviews, we explained the general objective of the research and clarified that their identities would be preserved. We continued with the interview only if the collaborator agreed to participate in the survey. Therefore, all collaborators orally confirmed free and informed consent prior to data collection.

### Visual stimuli and projective interview

**Fig. 2:**
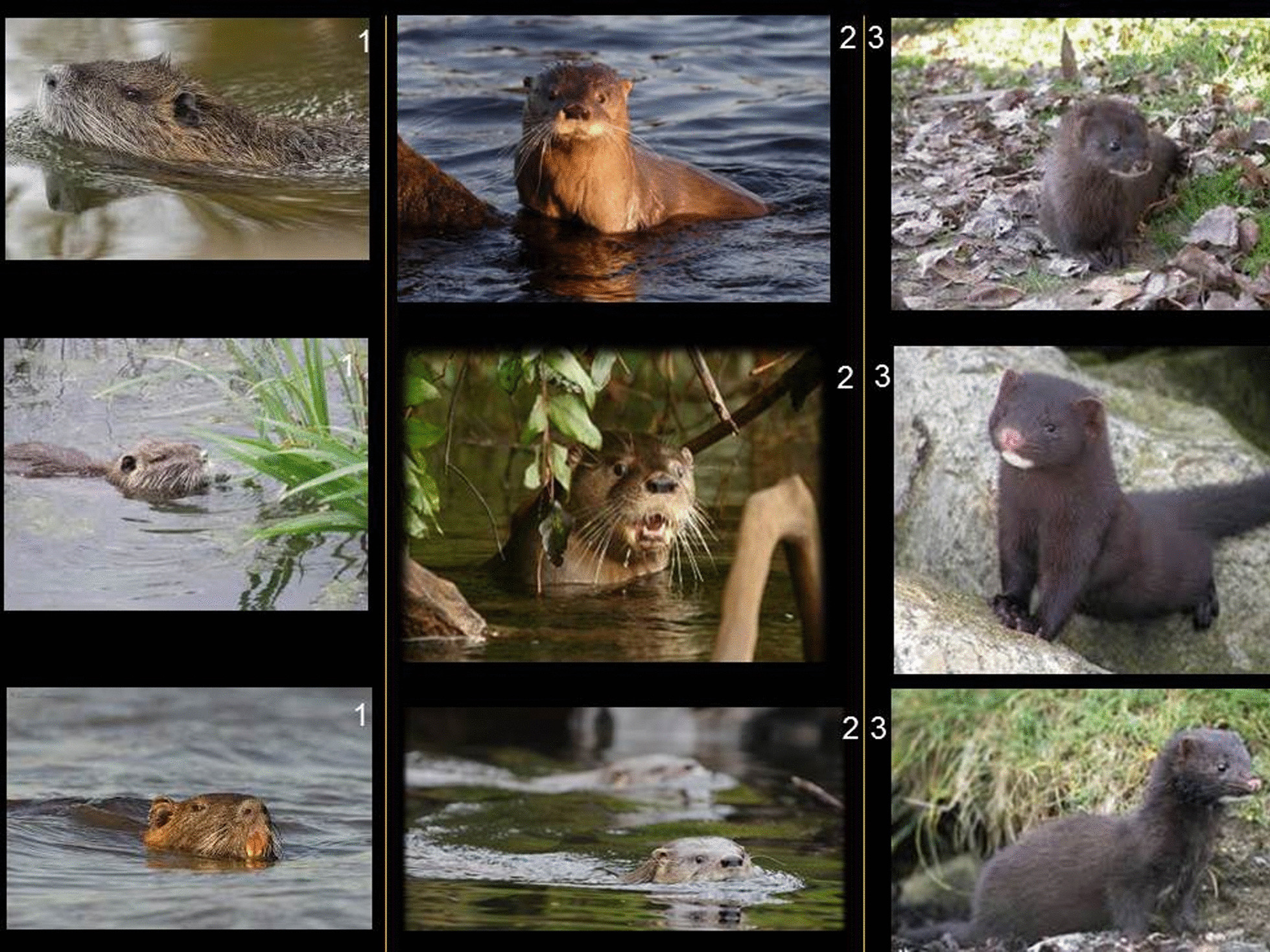
Visual stimulus. Image of the three semi-aquatic mammals that inhabit PNNH and the urban centers bordering the protected area. Column 1: coipo *(Myocastor castor),* Column 2: huillín *(Lontra provocax)* and Column 3: american mink *(Neovison vison)*

Prior to the workshop, a written projective interview was conducted with the participants. Visual stimuli were used to analyze LZK and to ensure that participants referred specifically to the huillín, but also to inquire about the LZK of other species with which the huillín may be confused. Good quality photos were used as visual stimuli and presented using a projector (Fig. 2). The stimulus panel had three columns: in Column 1 was placed the coipo, in Column 2 the huillín and in Column 3 the american mink. Photos of the animals in different positions and in which most of their distinctive features were visible were selected in order to help characterize them. It should be noted that visual stimuli are increasingly used in ethnozoological research [21, 22, 47–49]. They are an auxiliary strategy to help people remember certain types of information that may be of interest or a means to contextually orient the interviewee by offering specific details of the species under study. Images, photographs or films are used [50].

Participants completed the survey individually, which also required information on age, gender, where they lived and how long they had lived in the area. They were especially asked if they knew the species located in columns 1, 2 and 3 of the projected image and what name they gave to each one. In addition, if they had ever sighted a huillín, in what place and when. In total, 96 written interviews were conducted, 42 with women and 54 with men, who identified themselves as such. The interview is available in Additional file 1: Figure Sa.

### LZK mapping

In a second step, participants who provided spatiotemporal information in the projective survey (date and place of encounter with a huillín) were personally contacted. Additional open-ended interviews were conducted to determine the exact georeference of the sites reported and then included in a map, using Geographic Information Systems (GIS) as a tool. This cartographic tool visually represents an approximation of the LZK about the huillín. In this map, records were placed by tourist guides, neighbors from San Carlos de Bariloche and staff from protected areas, in combination with previous information obtained from official sources. As a reliability criterion, only people who were able to effectively distinguish the huillín in the written interview were taken into account. It should be noted that some people indicated a specific sighting site and others named the water body “at a general level”; references were placed on the map in both cases. When people named lakes, ponds or streams “at a general level,” a georeference was placed in the center of the water body. In cases where points were repeated, they were placed side by side. GIS is an increasingly relevant instrument for decision-making in biodiversity conservation [51], defined as a set of tools for collecting, storing, extracting, transforming and displaying “key” real-world spatial data for land management [52]. Among these particular purposes is to support land use planning and management processes as well as species conservation [51, 53, 54].

### Data analysis

Data analysis was quali-quantitative [46, 55]. First, a database was organized and restructured according to missing data, mainly regarding participants age and years of permanence at the site. In total, 92 records were considered. For the assignment of the local names of the three species, these were recorded directly from the interviews, but matches with the regional reference bibliography were then surveyed, assigning two categories of accuracy: positive (1) and negative (0). The names in the bibliography for the species in question are shown in Additional file 1: Table Sb. Normally, people gave only one name for the species, but in the cases where two or more names were given, it was considered “positive (= 1)” when at least one of the names coincided with one of those cited in Additional file 1: Table Sb. In the case of the huillín, when people answered “river otter” or “Patagonian otter,” it was categorized as positive.

The analyzed variables and categorization criteria are shown in Table 1. The possible association or independence between categorical variables was analyzed by means of contingency tables, using the chi-square statistic (χ2). In addition, three models were developed: (a) binomial logistic regression for huillín identification (Model 1), (b) binomial logistic regression for huillín sighting (Model 2) and (c) ordinal logistic regression for identification of semi-aquatic mammals (Model 3). The aim was to evaluate the probability that these dependent variables vary according to the sociocultural characteristics of the people interviewed.

**Table 1:** Categorization of dependent and independent variables of this study

Variables	Variable type categorical	Categorization
huillín identification	binary	Yes/No
huillín observation	binary	Yes/No
coipo identification	binary	Yes/No
american mink identification	binary	Yes/No
Degree of knowledge of semiaquatic mammals	Multistate	No/ 0/1/2/3 (coincidence)
Social group	Multistate	Tour guides, Protected areas staff, Bariloche residents
Ambit	binary	Urban/rural
Age	Multistate	> 30 /30–60/ < 60
Time spent on site	Multistate	> 20/20–40/ < 40
Gender	Multistate	F/M/X

**Model 1.** P (Huillín identification) = β0 + β1 (Social group) + β2 (Setting) + β3 (Age) + β4 (Gender) + β5 (Permanence).

**Model 2.** P (Huillín sighting) = β0 + β1 (Social group) + β2 (Setting) + β3 (Age) + β4 (Gender) + β5 (Permanence).

**Model 3.** P (Degree of knowledge) = β0 + β1 + β2 + β3 (Social group) + β4 (Setting) + β5. (Age) + β6 (Gender) + β7. (Permanence).

A significance level less than or equal to 0.05 (*p* ≤ 0.05) was established for all tests. The link function used was: g(*μ*i) = ln(*μ*i/(1-*μ*i)), where *μ*i is the mean response of the ith row. For the statistical analyses, we used the IBM® SPSS and R programs (brglm2 package (Bias Reduction in Binomial-Response Generalized Linear Models).

In our study, we used QGIS to visualize, create and analyze spatial data about people's knowledge of the Huillín [56, 57]. Satellite images obtained from different databases and processed with this program were used as a cartographic base. We obtained a knowledge map in which information layers were added, such as pre-existing huillín locations published in the Biodiversity Information System of the Administración de Parques Nacionales (https://sib.gob.ar/portada) and the new dataset.

### Methodological limitations of the study

The results found could be biased by some methodological limitations. Mainly, we do not know the degree to which people are familiar with each species; their simple recognition is only one of the aspects that make up LZK and, undoubtedly, people must know differentially distinct aspects of the species. On the other hand, the statistical models used may be strongly affected when the categorical variables are not equally distributed or when some categories of variables have few sightings.

## Results

### Huillín identification

The different people who attended the workshops and participated in this study had a mean age of 40.1 ± 12.7 years. Out of the total number of participants, 95% identified the huillín, 3% confused it with the coipo and 5% with the american mink. The species was named as “huillín,” “bullin,” “lobito de rio” and “nutria,” which are the local names historically used according to the bibliography.

In contrast to our prediction, our results show that huillín identification did not vary with the social groups interviewed (χ2, *p* = 0.834). Positive identification was not affected by any variable, with the exception of people with permanence between 20 and 40 years, who identified the huillín less than those with permanence of less than 20 years; however, this tendency is marginally significant (z-value: − 2.004; *p* = 0.2691, Table 2).

**Table 2:** Binomial logistic regression model, huillín identification

	Estimate	Std. Error	Z value	Pr( >|z|)
(Intercept)	20.2985	1913.6189	0.011	0.9915
Setting “urban”	2.1409	1.1975	1.788	00,738
Gender “male”	0.1924	0.8544	0.225	0.8218
Protected areas staff	− 1.2615	1.1737	− 1.075	0.2825
Neighbour	− 0.6810	1.1099	− 0.614	0.5395
Age 30–60	− 17.3579	1913.6185	− 0.009	0.9928
Age < 60	− 19.1514	1913.6188	− 0.010	0.9920
Permanence 20–40	− 2.7163	1.3552	− 2.004	0.0450*
Permanence < 40	− 1.9104	1.7288	− 1.105	0.2691

Probability of huillín identification (yes/no) (dependent variable), according to the (independent) variables: setting, gender, social group (tourist guides, staff of protected areas, neighbors from San Carlos de Bariloche), age and permanence. The “Estimate” column shows the parameters of the generalized linear regression (GLM) for each independent variable (beta). The reference categories are: setting “rural,” gender “female,” social group “tourist guides,” age “ > 30 years,” permanence “ > 20 years.”

(*) Significant results in the model

### Huillín sighting

Similarly, in contrast to our prediction, the huillín sighting did not vary with the social groups interviewed (χ2, *p* = 0.898). Huillín sighting did not vary with any variable, with the exception of setting and permanence (z-value = − 2.920, *p* = 0.0032; z-value = 2.307, *p* = 0.0211, respectively, Table 3). Our results show that people from rural settings are 84% more likely to sight huillín than people from urban settings. In addition, people with permanence between 20 and 40 years have a probability of sighting huillín 3.80 times higher than those with less than 20 years of permanence.

**Table 3:** Binomial logistic regression model, huillín sighting

	Estimate	Std. Error	Z value	Pr( >|z|)
(Intercept)	− 0.35439	0.72208	− 0.491	0.6236
Setting “urban”	− 1.81850	0.62282	− 2.920	0.0035 **
Gender “male”	− 0.07268	0.50393	− 0.144	0.8853
Protected areas staff	0.83065	0.67240	1.235	0.2167
Neighbour	0.40273	0.63878	0.630	0.5284
Age 30–60	0.01894	0.57143	0.033	0.9736
Age < 60	0.93273	1.08987	0.856	0.3921
Permanence 20–40	1.33677	0.57948	2.307	0.0211 *
Permanence < 40	1.90634	1.09932	1.734	0.0829

Probability of huillín sighting (yes/no) (dependent variable), according to the (independent) variables: setting, gender, social group (tourist guides, staff of protected areas, neighbors from San Carlos de Bariloche), age and permanence. The “Estimate” column shows the parameters of the generalized linear regression (GLM) for each independent variable (beta). The reference categories are: setting “rural,” gender “female,” social group “tourist guides,” age “ > 30 years,” permanence “ > 20 years.”

(*) Significant results in the model

Our results show that 39% of the participants sighted a huillín and 61% never sighted one. A 58% of the positive sightings correspond to tourist guides, 22% to neighbors from San Carlos de Bariloche and 20% to staff from protected areas.

### Semi-aquatic mammal identification

In reference to the identification of semi-aquatic mammals distributed in Northern Patagonia, our results show that it does not vary with the social groups interviewed (χ2, *p* = 0.180). Furthermore, in relation to the identification of all semi-aquatic mammals, none of the variables studied was affected by the variables analyzed (Table 4, *p* > 0.05). Therefore, the proportion of people who identified semi-aquatic mammals does not vary with the sociocultural characteristics recorded.

**Table 4:** Ordinal logistic regression model, identification of semi-aquatic mammals inhabiting northern Patagonia

	Estimate	Std. Error	Z value	Pr( >|z|)
1: (Intercept)	− 1.2250	0.5969	− 2.05	0.040*
2: (Intercept)	− 0.0513	0.4493	− 0.11	0.909
3: (Intercept)	0.0621	0.4435	0.14	0.889
Age 30–60	− 0.3613	0.2804	− 1.29	0.198
Age < 60	0.0146	0.5482	0.03	0.979
Setting “urban”	− 0.5571	0.3044	− 1.83	0.067
Protected areas staff	− 0.2558	0.3767	− 0.68	0.497
Neighbour	0.2010	0.3198	0.63	0.530

Probability of identification of semi-aquatic mammals (yes/no) (dependent variable), according to the variables (independent): setting, gender, social group (tourist guides, staff of protected areas, neighbors from San Carlos de Bariloche), age and permanence. The “Estimate” column shows the parameters of the generalized linear regression (GLM) for each independent variable (beta). The reference categories are: age “ > 30 years,” setting “rural,” social group “tourist guides.”

(*) Significant results in the model

The coipo, huillín and american mink are species that are confused with other mammals and with each other. In the case of the coipo, 58% of the people interviewed could identify it and 42% could not, either because they did not know it (13%) or because they confused it with other mammals: capybara (*Hydrochoerus hydrochaeris,* Linnaeus 1766) (1%), beaver (*Castor sp*.) (23%), american mink (1%), huillín (3%), musk coipo (1%). In this last case, the name “musk coipo” does not coincide with any species registered in the regional reference bibliography consulted. It is possible that it refers to the muskrat *(Ondatra zibethicus,* Linnaeus, 1766).

In the case of the american mink, 63% of the people interviewed were able to identify it and 37% were unable to do so, either because they did not know it (13%) or because they confused it with other mammals: beaver (3%), huillín (5%), lesser grison (*Galictis cuja*, Molina 1782 or *Lyncodon patagonicus*, de Blainville 1842) (10%), otter (*Myocastor castor,* Molina 1782 or *Lontra provocax)* (3%), ermine mink (1%), European mink (1%), little bush monkey (*Dromiciops gliroides,* Thomas 1894) (1%). The name “stoat mink” does not coincide with any species recorded in the regional reference bibliography consulted. The stoat (*Mustela erminea,* Linnaeus 1758) is a species belonging to the same family as the american mink; it is a carnivorous mammal native to Europe. Possibly, they have referred to this species.

### LKZ mapping

**Fig. 3:**
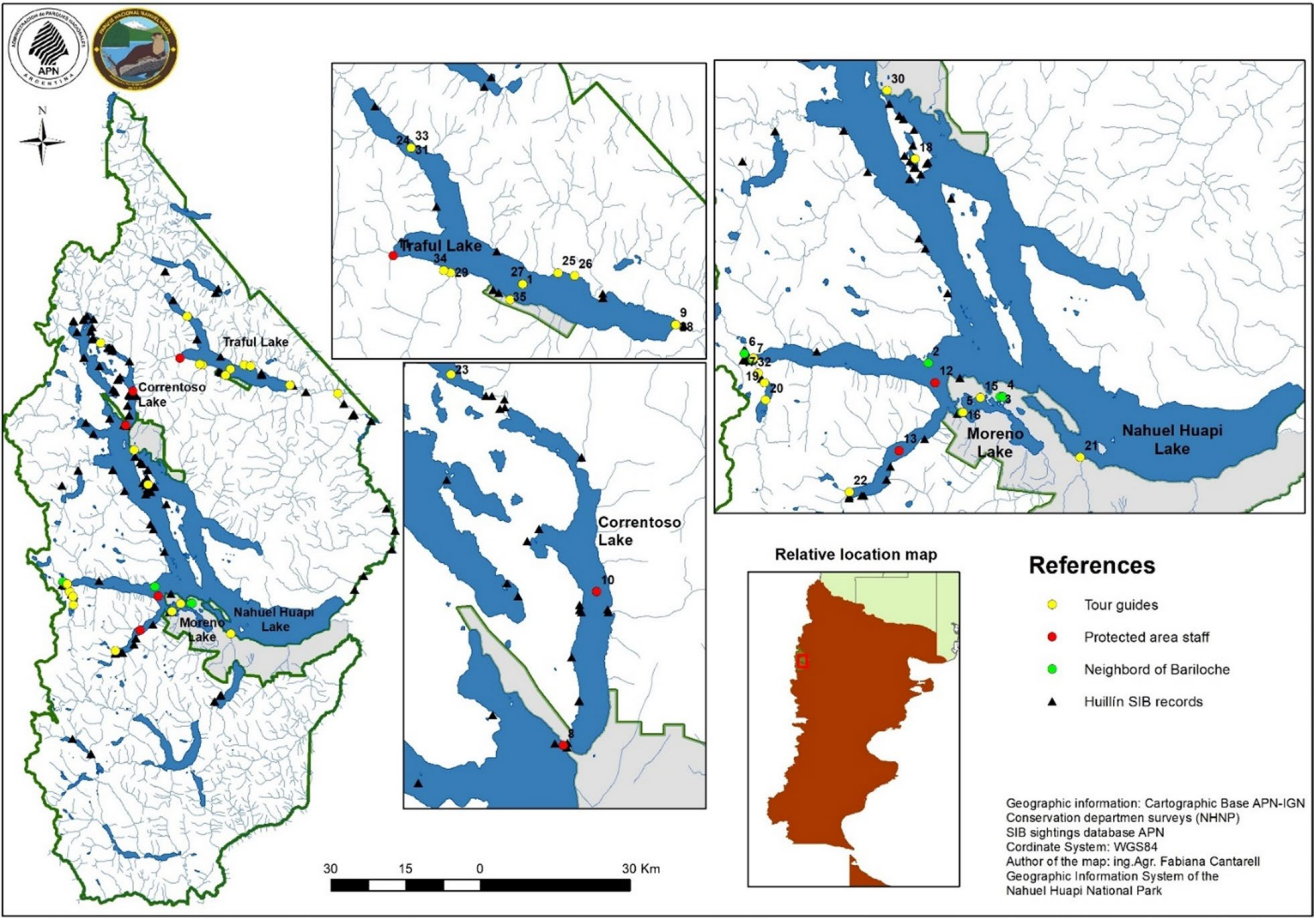
Huillín sightings recorded by people with diverse socio-cultural characteristics. These records were numbered on the map (1–36) and their geo-reference is found in Additional file 1: Table Sc

People identified 61.1% of the areas coinciding and/or adjacent to those recorded by official sources. They also identified 38.9% of other “non-coincident” areas that might be providing new information and represent new areas to explore (Fig. 3). To the west of Nahuel Huapi Lake and in Traful Lake there are areas with a concentration of points; this might be showing sites with medium/high magnitude of use by the huillín.

## Discussion

In general, LZK about the huillín did not vary significantly with the participants' socio-cultural characteristics, showing a remarkable homogeneity. These results could be a consequence of the updating, training and dissemination workshops previously provided to PNNH tour guides, staff of national, provincial and municipal protected areas, and the community in general, framed within the Protected Area Management Plan approved in 2019. In addition, the huillín has been the main focus of local outreach campaigns that could be key to ensure that people identify the species and have the necessary tools to recognize and sight it.

According to [58], variation and heterogeneity in ethnobiological knowledge is a significant aspect for adaptability in socio-environmental systems that are undergoing severe changes, as is our case. However, although it may seem paradoxical, the homogeneity found in the knowledge about huillín would show that it has been “fixed” within the population, possibly through oblique transmission routes (via workshops) and/or other modes of intra-familial cultural transmission not studied in the present paper.

In a study with fishermen from Ciénaga Grande de Santa Marta (Colombia), detected a similar situation regarding the LZK about crabs (*Callinectes sapidus,* Rathbun 1896) and *C. bocourti,* Edwards 1879) [59]. These authors suggest that the homogeneity of LZK represents a baseline to promote further conservation actions. Similarly, in our work, although it is necessary to carry out studies that include other sectors of society, the homogeneity found would suggest that there is also a solid baseline that would allow the development of effective conservation strategies together with local communities, especially with tourist guides, neighbors, and protected area staff.

Our results show that people from rural settings are more likely to sight the species than people from urban settings. This pattern has also been found in other studies [60–63] that show the effects of direct experiences with nature. The rural setting is, in general, in a better state of conservation than the urban setting in terms of habitat requirements for the huillín, that is, coastal vegetation cover and water quality. This is consistent with monitoring carried out in and around PNNH, where a greater number of positive huillín records were found in rural areas than in urban areas [44, 64]. On the other hand, the rural setting is a social space where, in general, people depend on agriculture, livestock, hunting, fishing, etc.; these activities generate processes of appropriation and learning about the setting and its co-inhabitants [65].

Several authors have shown that ethnobiological learning is episodic; it depends on a triggering event that generates in individuals the search for answers within their social environment of reference [66]. In the case of the huillín this is key, given that it is an elusive species. Therefore, we understand that in a rural setting these mechanisms can occur more easily. On the contrary, in the urban setting of San Carlos de Bariloche, the coastal and aquatic environments are anthropically disturbed, a fact that decreases the probability of sightings and thus decreases the probability of learning from experience and interaction with the environments.

Our work highlighted that people's permanence in the area for 20 to 40 years is an important factor in recognizing the huillín. Several studies have shown that the longer a person stays in the same place, the greater the opportunities to incorporate LZK, that is, to learn and appropriate the environment [67]. However, in our case, we did not find this relationship with people’s longer or shorter lengths of residence. These results could be interpreted in light of [68], who suggest that the ethnobiological knowledge of fishermen cohabiting with fish species stabilizes at approximately 30 years of age; at older ages, there are no major differences. We can then consider that this time span from 20 to 40 years is key for the appropriation of LZK about the huillín through individual learning and/or through what was learned in the PNNH workshops.

The participants who attended the workshops and reported having sighted the huillín were people who live and/or work close to the species range (53%) and/or within it (47%), but who do not spend 100% of their time in activities that require them to remain in aquatic environments. It could be that the variable “time spent in aquatic environments” where this species lives is a more appropriate variable than the “time of permanence in the place” that we used. This has been shown by studies that have investigated the LZK about otters in diverse Latin American communities [21, 22].

In agreement with [69] studies should be carefully designed to measure the interactive effect of age, time of residence, gender, etc. on local knowledge dynamics. In our case, our models were not significant considering the interactions, but they make clear that beyond analyzing these variables quantitatively, it is important to delve into the complex mechanisms and processes that generate or not patterns of knowledge based on age-time of residence or gender.

On the other hand, 94.57% of people were able to identify the huillín and few people (5.43%) confused it with other semi-aquatic mammals that inhabit the same environments, showing that among the participants the LZK about the huillín is an important part of their body of environmental knowledge. However, given that the workshops have been voluntary, it is very likely that this has been biased towards people particularly interested in the species, anyway demonstrating the success of this type of proposal.

The identification of semi-aquatic mammals inhabiting northern Patagonia, in general, did not vary significantly with the participants' sociocultural characteristics, showing a remarkable homogeneity. However, confusions were observed in the identification among coipo, huillín and american mink, which would indicate specific limitations of their LZK that should be taken into account in future studies. Difficulties of this type have been described in bat species, where similar morphological features make it difficult to correctly identify *Myotis keaysi*, Allen 1714 and *M. nigricans,* Schinz 1821, for example [70]. The coipo, huillín and american mink were named after other mammals with which they may share morphological and/or ethological traits but in some cases are very distant in taxonomic location, such as the beaver and its confusion with the huillín and the american mink.

On the other hand, the american mink was confused with the ferret (10%). This could be due to the fact that they have similar morphological and ethological characteristics, since they belong to the same subfamily (Mustelidae). These results may serve as a warning regarding the invasion processes of the american mink and the importance of working with citizens. For the NHNP, the invasion of exotic species is a serious problem that threatens the conservation of the natural, cultural, and social values of the protected areas under its management.

The low number of people who were able to sight huillínes (36%) evidences how challenging it is to record this species. Otters are elusive and cryptic animals, mimetic and with large territories, which translates into a low and difficult probability of sighting [21, 38]. Acquiring reliable estimates for the distribution and population size of an elusive species from scientific knowledge alone can be problematic [40]. Complementing with other methods and using approaches such as citizen science allows for the construction of recording networks and collective knowledge that could be “key” for the conservation of the huillín. In addition, it could increase public awareness towards its conservation, a positive trend that has been recorded in the study of other otter species [38]. Citizen science programs that truly monitor species perceived as charismatic or ecologically significant in a participatory manner tend to involve a larger group of potential volunteers with greater long-term commitment [36].

The concentration of recording points in specific areas of the map achieved in this work could be showing areas of high value for the species, such as active corridors, location of resting places, “key” feeding areas, etc. In an interesting investigation [39] the concentrations of positive sites are called "hot spots" and they are described as the sites with the best chances of finding otters.

## Conclusions

The biocultural importance of the huillín for the Patagonian region and its people is gradually being made visible [71]. With this participatory work we realize that the species is recognized especially by urban and rural inhabitants, since very few confuse it with other species. The homogeneity in LZK found on this species is a starting point for other participatory studies of greater depth. It is a relevant approach in the PNNH and surrounding area and promotes lines of research, action and management to improve the quality of the environments inhabited by the only known and stable freshwater population of huillín in Argentina.

The huillín is not only a charismatic species, but it is also considered an “umbrella” species [3], so this type of approach has a value that goes beyond the huillín, including the conservation of habitats and habits of all its co-inhabitants. This reorientation of joint work between scientists and people is essential. It implies the mutual sharing of benefits in knowledge, learning and valuing the environment where the huillín co-inhabits.

## Supplementary information

**Additional file 1.** Supplementary Figure and Tables.

## Data Availability

The data used to support the findings of this study are available from the corresponding author upon reasonable request.
