# Supplementary material for: Variation of local zoological knowledge about Southern river otter and other semi-aquatic mammals in Nahuel Huapi National Park (Argentina)
Source: J Ethnobiol Ethnomed. 2023 May 9;19:15. doi: 10.1186/s13002-023-00590-8 (PMC10170775; doi:10.1186/s13002-023-00590-8)
Supplement: Supplementary file 1 — Additional file 1. Supplementary Figure and Tables. [file 13002_2023_590_MOESM1_ESM.docx]

**Variation of local zoological knowledge about Southern river otter and other semi-aquatic mammals in Nahuel Huapi National Park (Argentina)**

Carla M. Pozzi¹ ³ and Ana H. Ladio² ³*

*Correspondence: [ahladio@gmail.com](mailto:ahladio@gmail.com)

¹ Área Biología de la Conservación, Programa de Estudios Aplicados a la Conservación del Parque Nacional Nahuel Huapi (CENAC), Depto. de Conservación y Educación Ambiental – Parque Nacional Nahuel Huapi, San Carlos de Bariloche, Río Negro, Argentina. ²Instituto de Investigaciones en Biodiversidad y Medio Ambiente (INIBIOMA) CONICET, Universidad Nacional del Comahue, San Carlos de Bariloche.³Grupo de Etnobiología - Universidad Nacional del Comahue, San Carlos de Bariloche, Río Negro, Argentina.

**SUPPLEMENTARY MATERIAL**

**Figure a.**

-Age: ..................................................................................................................................

- Email address (complete if you registered a huillín): .............................................................................................................................................

-In which neighborhood do you currently live?.......................................................................................

-¿ How long has he lived all?.......................................................................................................................................

**1-** The images on the screen have the number 1, 2 and 3. Each number corresponds to a different species. What do you call the animal presented in the images with the number 1, 2 and 3?

1……………………………

2……………………………

3……………………………

**2-** a) Have you ever observed a huillín?

SI…….
NO…...

b) Where did he observe it? (if you can give site details)

………………………………………………………………………………………………………………………………………………………………………………………………………………………………………………………………………………………………

c) When I look at it?

- This year (2019)………..

- Another year which:……..

-¿ Remember the season of the year?

Autumn………
Winter…….
Spring…..
Summer...

Interview with different social groups linked to Nahuel Huapi National Park.

**Table a**.

| **Number** | **Workshop year** | **Title** | **“Summoned Social Group”** |
| --- | --- | --- | --- |
| 1 | 2015 | Sharing knowledge about the huillín, an otter with which we live in our neighborhoods. | NEIGHBOR OF BARILOCHE |
| 2 | 2016 | Update of knowledge of the fauna of the PNNH | PNNH TOUR GUIDES |
| 3 |  | Registration of the distribution of the huillín in the PNNH | PROTECTED AREAS STAFF (Park rangers of the PNNH and Rangers of the province of Neuquén) |
| 4 | 2017 | Update of knowledge of the fauna of the PNNH | PNNH TOUR GUIDES |
| 5 | 2018 | Update of knowledge of the fauna of the PNNH | PNNH TOUR GUIDES |
| 6 |  | Knowledge about the semi-aquatic mammals that are distributed in Northern Patagonia. | PNNH TOUR GUIDES |

Participatory workshops with people of diverse socio-cultural characteristics linked to PNNH.

**Table b.**

| **Scientific name** | **Common names** | **Bibliography** |
| --- | --- | --- |
| *Myocastor castor* | coipo, nutria | -Canevari y Vaccaro 2007 |
| *Lontra provocax* | huillín, nutria | -Villagrán 1999,  -Canevari y Vaccaro 2007,  Mocuhard 2019) |
|  | bullín | -Chehébar y Ramilo 1992 |
|  | lobito de rio patagónico | -Chehébar y Ramilo 1992  -Iriarte y Jaksik 2012 |
|  | lobito patagónico | -Mocuhard 2019 |
| *Neovison vison* | vison, vison americano | -Bonino 2005  -Canevari y Vaccaro 2007 |

Existing names in the literature for the species of semi-aquatic mammals of northern Patagonia.

**Table c.**

| **N°** | **lat in decimal degrees** | **long in decimal degrees** | **Social Group** | **Interview number** |
| --- | --- | --- | --- | --- |
| 1 | 40,63472222 | 71,42667 | Neighbor of Bariloche | 4 |
| 2 | 41,02742778 | 71,60381 | Neighbor of Bariloche | 10 |
| 3 | 41,05761389 | 71,51767 | Neighbor of Bariloche | 13 |
| 4 | 41,05739167 | 71,51439722 | Neighbor of Bariloche | 17 |
| 5 | 41,07174167 | 71,56346111 | Neighbor of Bariloche | 22 |
| 6 | 41,01919167 | 71,82304167 | Neighbor of Bariloche | 22 |
| 7 | 41,024725 | 71,81361389 | Neighbor of Bariloche | 22 |
| 8 | 40,73676389 | 71,67478889 | Protected Area Staff | 27 |
| 9 | 40,66265833 | 71,28506944 | Protected Area Staff | 30 |
| 10 | 40,67495278 | 71,65761944 | Protected Area Staff | 32 |
| 11 | 40,61535278 | 71,546225 | Protected Area Staff | 32 |
| 12 | 41,044975 | 71,59514722 | Protected Area Staff | 33 |
| 13 | 41,10641667 | 71,63801944 | Protected Area Staff | 33 |
| 14 | 41,02325 | 71,81239722 | Protected Area Staff | 38 |
| 15 | 41,05816389 | 71,54143889 | Tourist Guides | 39 |
| 16 | 41,07171389 | 71,56216667 | Tourist Guides | 41 |
| 17 | 41,03688333 | 71,80707222 | Tourist Guides | 42 |
| 18 | 40,84280556 | 71,62065833 | Tourist Guides | 43 |
| 19 | 41,04555556 | 71,79918056 | Tourist Guides | 44 |
| 20 | 41,06110556 | 71,79785278 | Tourist Guides | 59 |
| 21 | 41,11156111 | 71,42165833 | Tourist Guides | 60 |
| 22 | 41,14393889 | 71,69739722 | Tourist Guides | 66 |
| 23 | 40,58821111 | 71,73478889 | Tourist Guides | 72 |
| 24 | 40,53948611 | 71,53018611 | Tourist Guides | 75 |
| 25 | 40,62677778 | 71,39390556 | Tourist Guides | 77 |
| 26 | 40,62875 | 71,37873056 | Tourist Guides | 77 |
| 27 | 40,63472222 | 71,42666667 | Tourist Guides | 78 |
| 28 | 40,66265833 | 71,28506944 | Tourist Guides | 79 |
| 29 | 40,62699722 | 71,49315833 | Tourist Guides | 87 |
| 30 | 40,78136389 | 71,65428056 | Tourist Guides | 87 |
| 31 | 40,53948611 | 71,53018611 | Tourist Guides | 87 |
| 32 | 41,02325 | 71,81239722 | Tourist Guides | 88 |
| 33 | 40,53948611 | 71,53018611 | Tourist Guides | 90 |
| 34 | 40,62565278 | 71,49928333 | Tourist Guides | 90 |
| 35 | 40,64571389 | 71,43810278 | Tourist Guides | 90 |
| 36 | 40,67667222 | 71,17170556 | Tourist Guides | 92 |

Numbering and geographic location of the sites registered by the various social groups in **Figure 3.**
